# Functionalized Nanoporous Biocarbon with High Specific Surface Area Derived from Waste Hardwood Chips for CO_2_ Capture and Supercapacitors

**DOI:** 10.1002/smsc.202500174

**Published:** 2025-06-24

**Authors:** Jibi Kunjumon, Ajanya Maria Ruban, Harleen Kaur, Davidson Sajan, Sanje Mahasivam, Vipul Bansal, Gurwinder Singh, Ajayan Vinu

**Affiliations:** ^1^ Global Innovative Centre for Advanced Nanomaterials (GICAN) College of Science, Engineering, and Environment (CESE) School of Engineering University of Newcastle Callaghan NSW 2308 Australia; ^2^ Department of Physics Centre for Sustainable Energy and Environmental Technologies (CE2T) Bishop Moore College Mavelikara Alappuzha Kerala 690110 India; ^3^ Sir Ian Potter NanoBioSensing Facility NanoBiotechnology Research Laboratory School of Science RMIT University Melbourne Victoria 3000 Australia

**Keywords:** CO_2_ capture, high surface areas, oxygen functionalization, porous carbons, supercapacitors

## Abstract

Waste biomass has aroused increasing interest in the production of low‐cost materials for CO_2_ adsorption and supercapacitors. One of the primary facets in this regard is to develop nanoporous carbons with controlled porosity and high surface area. Herein, waste wood chips are used to synthesize nanoporous biocarbons via a solid‐state KOH‐based chemical activation. The synthesized materials presented high surface area (3686.10 m^2^ g^−1^), large pore volume (1.88 cm^3^ g^−1^), and tunable pore sizes. As a porous solid adsorbent, the optimized material adsorbs 5.59 mmoles of CO_2_ per gram at 0 °C/1 bar, which is elevated to 37.47 mmoles g^−1^ at 0 °C/30 bar along with a good CO_2_/N_2_ selectivity within a range ≈25–35 and also displays high recyclability of >99%. Electrochemically, in a three‐electrode setup, a high specific capacitance of 261.5 F g^−1^/0.5 A g^−1^ is observed. For a two‐electrode setup, a reasonable specific capacitance of 91.67 F g^−1^/0.5 A g^−1^, energy and power densities (18.33 Wh kg^−1^ and 2274.94 kW kg^−1^), and 87.5% capacity retention after 10 000 cycles are obtained. A low‐cost and noncomplicated synthesis and high performance of materials for CO_2_ adsorption and supercapacitors make a strong case for their high promise in these fields.

## Graphical abstract

The presented article highlights the facile conversion of waste wood chips into high surface area and surface functionalized porous carbon materials via an effective KOH‐based solid‐state chemical activation and their utilization as highly efficient adsorbents for CO_2_ capture and as electroactive electrode materials for three and two electrode supercapacitors.

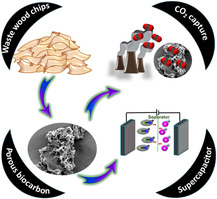

## 1 Introduction

The need to reduce greenhouse gas (GHG) emissions to control climate change and protect the environment is becoming more widely understood.^[^ ^1^ ^]^ Among GHGs, carbon dioxide (CO_2_) is the most lethal in its impact on global warming due to its longer residence time in the atmosphere.^[^ ^2^ ^]^ Anthropogenic sources of CO_2_ emissions, primarily including industrial sector‐based point sources, are major contributors, and a large focus is centered on combating emissions from such industries by developing and utilizing innovative technologies. One of the highly regarded technologies is CO_2_ capture using different types of methods, which could help minimize CO_2_ emissions and contribute towards sustainability. The second prominent issue of the current era is to shift focus from fossil fuels to more renewable technologies. In this domain, energy storage devices have a critical role to play. Supercapacitors are devices that are capable of storing large energy quantities and delivering high power density, which is desired in many energy sectors. The efficiency of supercapacitors relies mainly on the electrode material, which is mostly composed of carbon. However, their stability, long‐term durability, and improvement in capacitance are some of the commonly encountered issues.

The CO_2_ capture process can be accomplished by using technologies based on absorption, adsorption, cryogenic distillation, and separation processes.^[^ ^3^ ^]^ Among these, the absorption using aqueous amine solvents is the most developed and commercially used technology. While this technology can remove 90% of the CO_2_ in a post‐combustion process, the major drawbacks, such as amine toxicity, thermal degradation, and high heat of regeneration, thwart its long‐term capabilities.^[^ ^4^ ^]^ These issues can be appropriately addressed by using adsorption‐based CO_2_ capture, which is mostly done by using porous solids.^[^ ^5^ ^]^ Organic frameworks, porous polymers, zeolites, and porous carbons are the leading contenders for this purpose.^[^ ^6^ ^]^ Porous carbons possess a robust physicochemical structure with remarkable efficiency for CO_2_ capture under various conditions.^[^ ^7^ ^]^ As adsorption mainly relies on porosity and specific surface area in carbon‐based materials, it is always an active area of research to pursue.^[^ ^8^ ^]^ Although porous carbons can be derived from literally any carbonaceous precursor through high‐temperature carbonization, the utilization of carbonaceous precursors such as waste biomass brings in the extra element of sustainability and low cost associated with it. Additionally, their synthesis is far easier than other porous materials, and a desired level of porosity can be achieved readily via the fine adjustment of experimental parameters. Moreover, their functionalized counterparts can uplift CO_2_ adsorption capacity due to a synergistic action of doping and porosity. For instance, *P*‐doped porous carbons derived from lotus petiole adsorbed 6.05 mmol g^−1^ of CO_2_ at 0 °C/1 bar,^[^ ^9^ ^]^ S‐doped D‐glucose derived porous carbons showed 4.65 mmol g^−1^ of CO_2_ adsorption at 0 °C/1 bar,^[^ ^10^ ^]^ N and S co‐doped porous carbon derived from a phenol formaldehyde resin adsorbed 6.38 mmol g^−1^ of CO_2_ at 0 °C/1 bar,^[^ ^11^ ^]^ and petroleum coke‐based N and S co‐doped porous carbon showed 5.08 mmol g^−1^ of CO_2_ adsorption at 0 °C and 1 bar.^[^ ^12^ ^]^ However, the functionalization of porous carbon may come as an added step to the synthesis.

Wooden biomass presents an interesting proposition in this regard as it possesses high carbon content (82.14–84.70%) and low ash concentration (3.28–4.61%).^[^ ^13^ ^]^ This is an ideal situation to exploit the high carbon content of such biomass into nanoporous biocarbons by utilizing high‐temperature chemical activation. However, it is critical to regulate the content and distribution of micro and mesopores in porous carbons to control the adsorption of CO_2_ in them during low and high pressures. From an energy storage point of view, porous carbons form an ideal platform to devise the electrode materials, as these possess good thermal and chemical stability, ample porous channels for charge storage and ion diffusion, and provision for manipulation of surface chemistry for enhanced wettability and performance.^[^ ^14^ ^]^ In addition, the amorphous structure and rough surface morphology are appealing factors for improving the electrochemical ability.^[^ ^15^ ^]^ It is well established that micropores (<2 nm) support the charge storage while the mesopores (2–50 nm) improve the ion diffusion.^[^ ^16^ ^]^ Therefore, like CO_2_ adsorption, pore structure regulation also forms a critical component for porous carbons for their suitability as supercapacitor electrodes.^[^ ^17^ ^]^ Supercapacitors are excellent devices to provide high power density and good cycling stability in the energy storage field.^[^ ^18^ ^]^ However, porous carbons still do have limitations as well in terms of low energy density, high resistance, and low conductivity. Nevertheless, it is possible to sort these issues out to a certain extent through suitable modifications of porous carbons, which is beyond the scope of this article. Most importantly, porous carbon materials have risen in status and have recently been utilized as host materials for advanced energy applications such as zinc iodine batteries.^[^ ^19^ ^]^

The motivation of the current work lies in the conversion of waste wood chips (WWCs) into porous carbons via the process of solid‐state chemical activation using potassium hydroxide (KOH) and utilizing them as CO_2_ adsorbents and supercapacitor electrodes. Despite its wide use, KOH‐based chemical activation is still the predominant method for producing high surface area porous carbon from biomass.^[^ ^20^ ^]^ Chemical activation induced high surface areas in the synthesized porous carbons, and the ratio of the micro and mesopores was readily controlled by varying the amounts of KOH used for impregnation. A high surface area (3686.10 m^2^ g^−1^) aided in delivering the adsorption of 37.47 mmoles of CO_2_ per gram of the material at 0 °C/30 bar. The low‐pressure CO_2_ adsorption, at 0 °C/1 bar, was recorded as 5.59 mmol g^−1^, which is comparable to existing materials. Heat of adsorption values (20.27–27.70 kJ mol^−1^) suggested that the interactions between porous carbons and CO_2_ are physical. As a supercapacitor electrode in a three‐electrode setup, a good specific capacitance (261.5 F g^−1^) at a current density of 0.5 A g^−1^ was observed, which sits reasonably well among the published literature. For instance, among biomass derived porous carbons, it is higher than the N‐doped carbon derived from potato residue (255 F g^−1^/0.5 A g^−1^),^[^ ^21^ ^]^ graphene‐based N‐doped porous carbon aerogel (197 F g^−1^/0.2 A g^−1^),^[^ ^22^ ^]^ 3D N‐doped porous carbon derived from Taro stem (236.4 F g^−1^/0.1 A g^−1^),^[^ ^23^ ^]^ and porous carbon derived from hibiscus sabdariffa fruits (194.50 F g^−1^/0.5 A g^−1^).^[^ ^24^ ^]^ For a two‐electrode configuration, the porous carbon delivered a capacitance of 91.67 F g^−1^ at 0.5 A g^−1^ along with reasonably good energy and power densities (18.33 Wh kg^−1^ and 2274.94 W kg^−1^) at 7 A g^−1^, respectively. Additionally, 87.5% capacitive retention can be attained even after 10 000 cycles. Overall, the proposed research is a low‐cost pathway to synthesize porous carbon with a dual purpose of addressing the gas capture and supercapacitor electrodes.

## 2 Results and Discussion

### 2.1 Powder X‐Ray Diffraction (PXRD) Analysis

**Figure 1:**
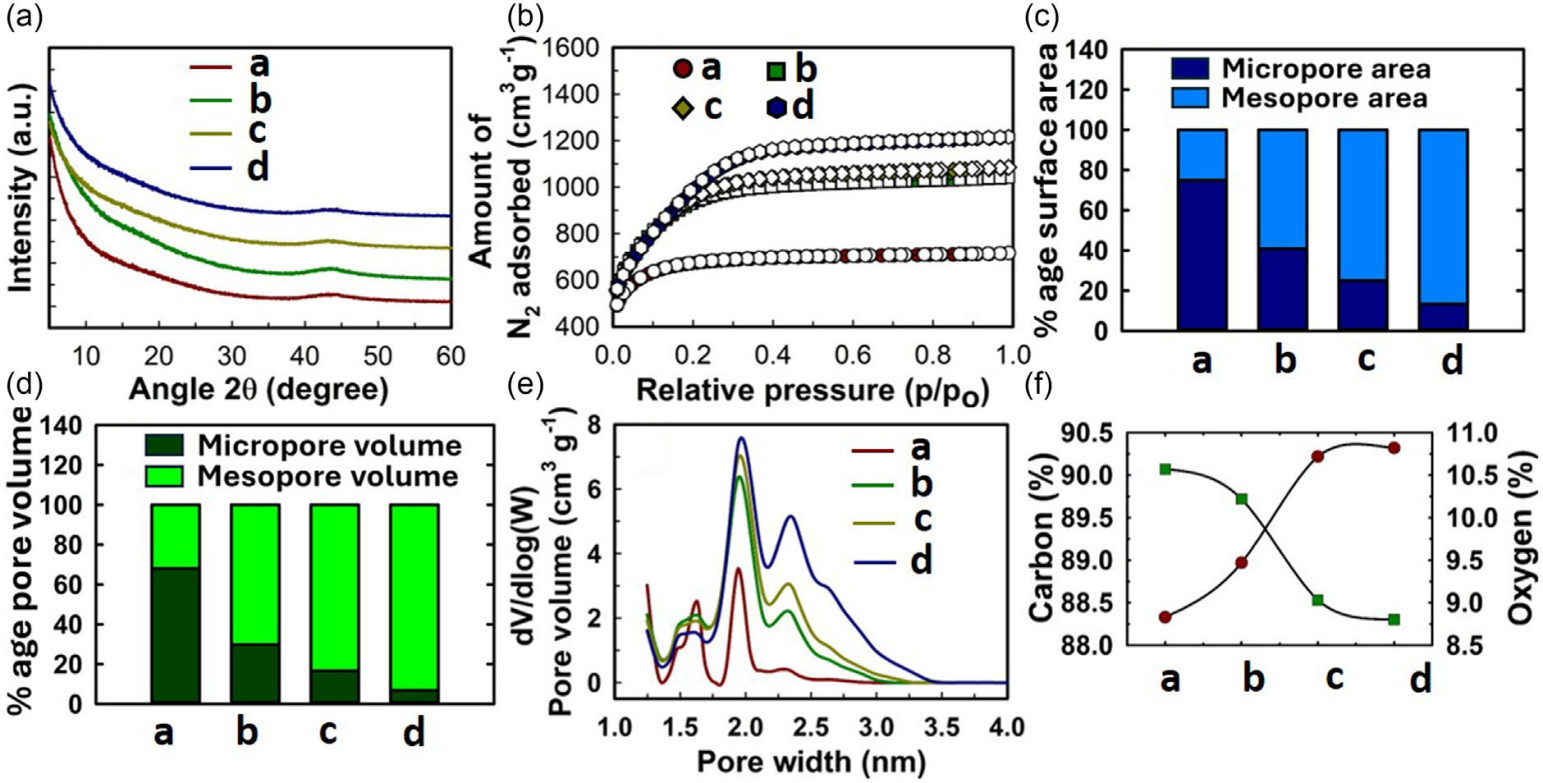
a) PXRD patterns, b) N_2_ sorption isotherms, c,d) percentage contribution from micropores and mesopores to surface area and pore volume, e) PSD, and f) carbon and oxygen weight percentage of the materials (where a = WPC600‐3, b = WPC600‐4, c = WPC600‐5, and d = WPC600‐6).

The crystallinity as well as the phase purity of the synthesized materials were assessed using PXRD analysis. The NPC materials, which were obtained using direct carbonization of WWCs at 400, 500 and 600 °C, showed the presence of crystalline peaks (Figure S2a, Supporting Information) that are presumed to be arising from various minerals accumulated during the growth period of the wood. The biomass, composed of carbon and other elements such as oxygen, nitrogen, and hydrogen, along with silica, on direct carbonization results in the loss of the majority of lighter noncarbon elements and thus forms a concentrated carbon network. Two broad humps around 2θ positions of 20.56° and 42.59°, represent the (002) and (100) reflections in an amorphous type carbon structure.^[^ ^28^ ^]^ NPC‐600 was further chemically activated with KOH using different impregnation ratios. The materials obtained with chemical activation (WPC600‐3,4,5,6) showed the presence of an amorphous carbon structure without the presence of any crystalline components, which indicates that KOH activation has effectively eliminated all impurities (**Figure** **1**a).^[^ ^29^ ^]^

### 2.2 Textural Parameters Analysis

KOH is a well‐known chemical activating agent to induce porosity in carbon‐rich precursors.^[^ ^30^ ^]^ During high‐temperature carbonization cum activation, KOH undergoes several redox reactions with carbon resulting in the formation of several products including metallic K, K_2_O, K_2_CO_3_, and volatiles including H_2_, CO, CO_2_, and H_2_O^[^ ^31^, ^32^ ^]^. In general, 800 °C is considered an optimal temperature for the full decomposition of KOH and to exert its maximum effect for chemical activation. During activation, the carbon framework gets etched by KOH, K_2_O, and K_2_CO_3_ to generate pores, escaping volatiles contributes to further porosity development, and the intercalation of metallic K between carbon layers helps to expand the carbon lattice. The acid washing frees up the intercalated metallic K and other inorganic residues to generate a high specific surface area containing a porous carbon framework.

Among porous carbons synthesized at various temperatures, WPC600‐4 presented a surface area of 3377.4 m^2^ g^−1^, whereas WPC400‐4 and WPC500‐4 showed lesser values of 3257.09 m^2^ g^−1^ and 3366.26 m^2^ g^−1^ although the shape of their sorption isotherms looks similar (Table S1 and Figure S2b, Supporting Information). This suggests that the materials possess a mix of micro and mesopores. The materials WPC600‐3,4,5,6 showed variation in their porosity based on N_2_ sorption analysis (Figure 1b). Qualitatively, the isotherm shape suggests that WPC600‐3 possess more of a microporous material type structure, which is evident from a high adsorption in the low‐pressure region below *p*/*p*_*o*_ of 0.1.^[^ ^33^ ^]^ The other materials show a gradual shift in their shape at low pressures, which indicates the development of micropores with higher pore sizes. This corroborates with porosity parameters obtained from quantification of the sorption data. The surface area of WPC600‐3 is the lowest (2332.90 m^2^ g^−1^), and it keeps on increasing with the increased amounts of KOH impregnation.

Among the nanoporous biocarbons prepared, WPC600‐6 presented the highest surface area of 3686.10 m^2^ g^−1^ (**Table** **1**). The microporous area showed a decreasing trend going from WPC600‐3 to WPC600‐6, as the higher amounts of activation tend to cause more etching of the carbon walls, which leads to the widening of existing micropores along with the formation of new mesopores (Figure 1c). Microporous and mesoporous volumes also displayed a similar trend (Figure 1d). The total pore volume of the materials showed an increasing trend, which was further confirmed through the creation of higher porosity by using larger amounts of KOH for activation. The nonlocal density functional theory (NLDFT)‐based pore size distribution (PSD) was evaluated using the obtained curves (Figure 1e) show a multimodal pore size spanning across a range of 1.5 to 3.0 nm. It is always challenging to control the PSD in KOH activation of biomass‐derived porous carbons due to the heterogeneity of biomass components and the aggressive nature of KOH. However, such systems are ideally conducive for several applications such as adsorption and energy storage, as micropores allow for higher adsorption and mesopores provide high mass transfer kinetics. The bulk elemental composition analysis of the materials revealed a predominant carbon content (≈90%) accompanied by oxygen (≈10%) (Figure 1f). The amount of KOH impregnation did not have a profound effect on their contents, except for a slight increase in carbon and a corresponding decrease in oxygen content while going from WPC600‐3 to WPC600‐6.

**Table 1:** Textural properties of the synthesized materials.

Material	SA_BET_ [m^2^ g^−1^]	SA_micro_ [m^2^ g^−1^]	PV_total_ [cm^3^ g^−1^]	*V*_micro_ [cm^3^ g^−1^]	PD [nm]
WPC600‐3	2332.90	1748.3 (74.9%)	1.10	0.75 (68.1%)	1.6/1.94/2.3
WPC600‐4	3377.94	1380.2 (40.8%)	1.61	0.48 (29.8%)	1.6/1.95/2.3
WPC600‐5	3487.60	872.8 (25.0%)	1.67	0.28 (16.7%)	1.6/1.96/2.3
WPC600‐6	3686.10	489.8 (13.3%)	1.88	0.13 (6.9%)	1.6/1.97/2.3

### 2.3 Scanning Electron Microscopy (SEM) cum Energy Dispersive X‐Ray (EDX) Analysis

**Figure 2:**
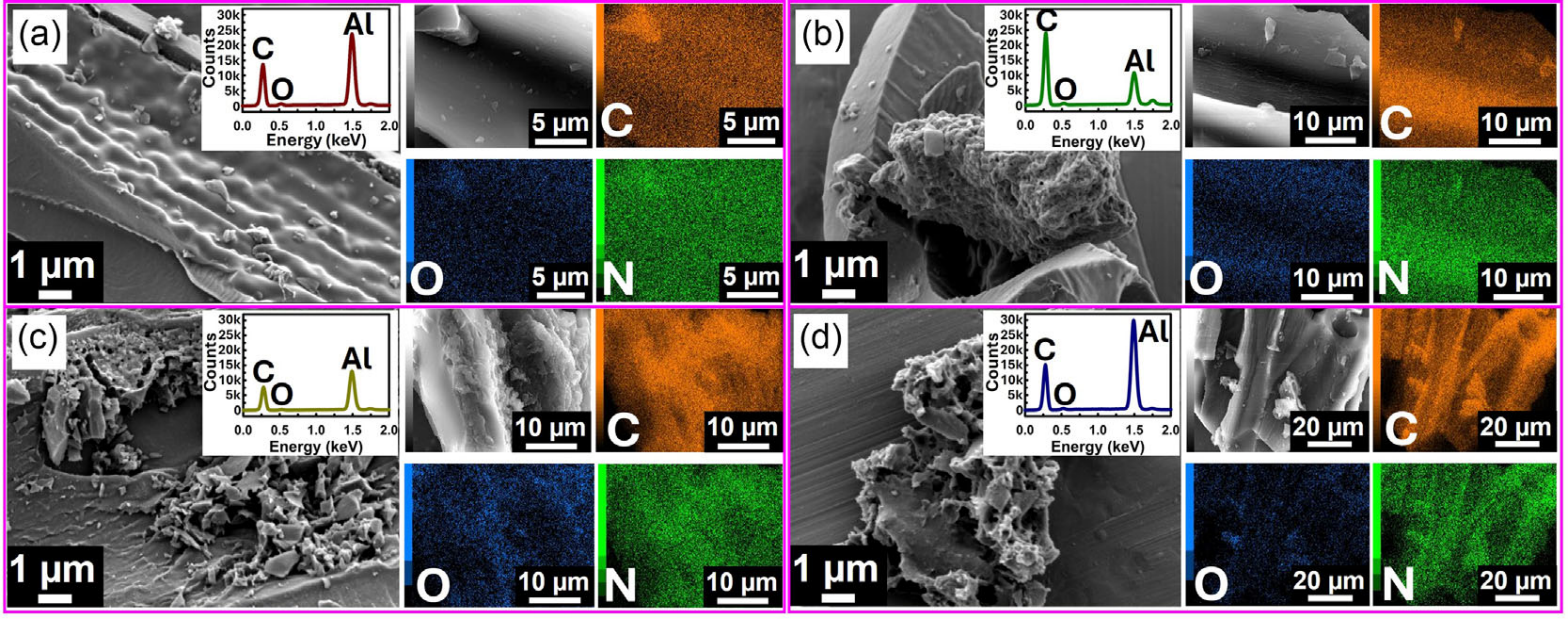
a–d) SEM images (EDX elemental quantification in the inset) and elemental mapping of WPC600‐3, WPC600‐4, WPC600‐5, and WPC600‐6.

The SEM images of WPC600‐3,4,5,6 are presented in **Figure** **2**. In general, the shape and size of the carbon particles are nonuniform and randomly distributed, which is a characteristic of an amorphous carbon type structure. The materials also underwent a change from a smooth surface with ridges in WPC600‐3 to a porous sponge‐type structure in WPC600‐4,5,6, which signifies the impact of the amount of KOH impregnation on the morphology of the materials.^[^ ^34^ ^]^ The degradation of biopolymers in wood chips upon high‐temperature carbonization cum KOH activation may lead to pore formation.^[^ ^35^ ^]^ The EDX‐based chemical composition reveals the predominant carbon nature of the materials, accompanied by oxygen (Figure 2a–d insets and Table S2, Supporting Information). The elemental mapping reveals a small distribution of nitrogen in materials other than carbon and oxygen (Figure 2a–d). A very small amount of nitrogen (<0.5%) was also detected in CHN analysis. However, being in very small amounts, XPS did not reveal any noticeable presence of nitrogen on the surface of the materials. The surface morphology of the materials synthesized at different temperatures indicates that at 400 °C, the structure is relatively smoother as compared to 500 °C or 600 °C (Figure S3, Supporting Information). The microstructure of WPC600‐6, which possesses the highest surface area, was further investigated using TEM imaging (Figure S4, Supporting Information), which revealed an amorphous structure composed of random carbon layers with a relatively thinner intensity on the boundary as compared to the bulk of the material.^[^ ^35^ ^]^

### 2.4 Fourier Transform Infrared (FTIR) Spectral Analysis

**Figure 3:**
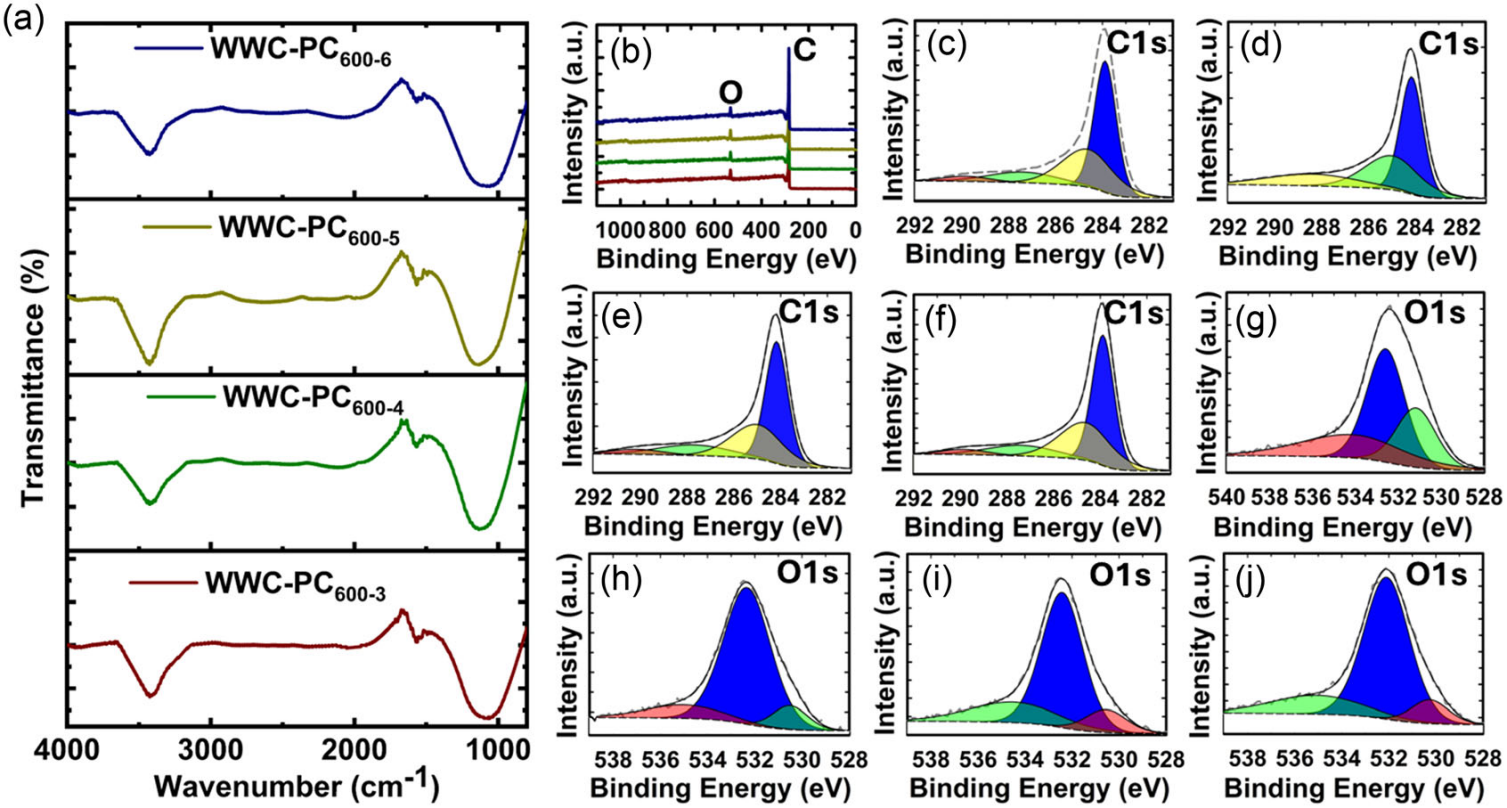
a) FTIR spectra, b) survey spectra (red—WPC600‐3, green—WPC600‐4, yellow—WPC600‐5, and blue—WPC600‐6), c–f) C1s high‐resolution spectra, and g–j) O1s high‐resolution spectra of WPC600‐3,4,5,6.

The surface functional groups in the WPC600‐3,4,5,6 were investigated using FTIR (**Figure** **3**a). The prepared samples show almost a similar appearance in all the materials with a predominance of carbon (C) and oxygen (O)‐containing functional groups. KOH activation can induce the development of surface oxygen functional groups during the process of carbonization cum activation. The O—H stretching vibrations were evident from a band observed at ≈3420 cm^−1^, whereas the position 1200–1100 cm^−1^ indicates C—O vibrations.^[^ ^36^ ^]^ The carbonyl stretching vibration (C=O) was observed ≈1560 cm^−1^.^[^ ^35^ ^]^ The oxygenated functional groups could potentially impart a negative charge, which could then be beneficial for enhancing the interactions with acidic CO_2_ molecules.^[^ ^29^ ^]^ Furthermore, such groups can contribute towards increasing the wettability of the material in electrochemical operations.^[^ ^37^ ^]^

### 2.5 X‐Ray Photoelectron Spectroscopy (XPS) Analysis

The surface elemental composition was evaluated using XPS analysis, and the results are shown in Figure 3b–j. The survey spectrum of WPC600‐3,4,5,6 showed peaks for carbon and oxygen (Figure 3b). In the survey spectrum, carbon was found to be the dominant element, which validated the carbonaceous behavior of the materials. The deconvoluted C1s spectrum of WPC600‐3,4,5,6 presents several peaks (Figure 3c–f). For WPC600‐5, the C1s spectrum is deconvoluted into four peaks corresponding to the binding energy positions 284.16 eV, 284.99 eV and 287.7 eV, and 290.12 eV, indicating sp^2^ hybridized carbon in C=C bond, C—O/C=O bonds, and O—C=O bonds, respectively.^[^ ^38^ ^]^ High‐resolution O1s spectra of the activated materials (Figure 3 g–j), wherein WPC600‐5 (Figure 3i) revealed three peaks located at ≈530.55 eV, 532.41 eV, and 534.49 eV indicative of carbonyl groups (C=O), C—O—C groups, and surface‐adsorbed oxygen or water.^[^ ^39^, ^40^ ^]^ These results correlate with the findings of FTIR.

## 3 Application Evaluation

### 3.1 CO_2_ Adsorption

**Figure 4:**
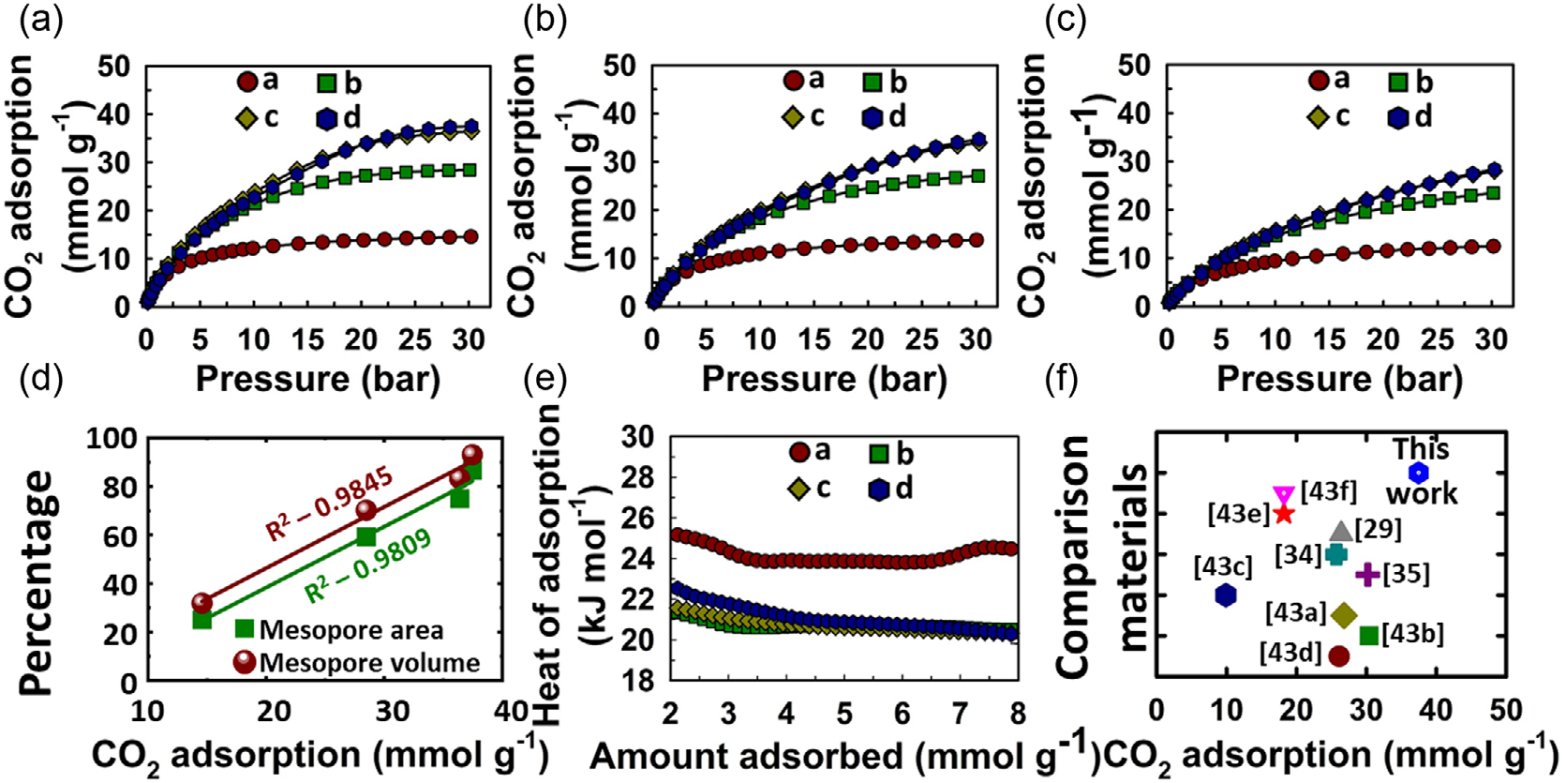
a–c) CO_2_ adsorption isotherms at 0, 10, and 25 °C (0–30 bar), d) linear regression fitting of the mesopore volume and area with CO_2_ adsorption at 30 bar, e) heat of adsorption of the materials (where a = WPC600‐3, b = WPC600‐4, c = WPC600‐5, and d = WPC600‐6), and f) comparison of the CO_2_ adsorption of presented material WPC600‐6 with the published literature.^[^ ^29^, ^34^, ^35^, ^43^ ^]^

Porous carbons are good contenders for high‐pressure CO_2_ adsorption and, with their tunable micropore structure, can also perform reasonably well at atmospheric pressures.^[^ ^41^ ^]^ The presented materials WPC600‐3,4,5,6 possess high surface areas along with adjustable pore features and hence were analyzed for CO_2_ adsorption within a wide pressure range of 0–30 bar (**Figure** **4**).

#### 3.1.1 Effect of Surface Area and Mesopore Content on High‐Pressure CO_2_ Adsorption

The surface area and the mesoporous content of the porous carbons play a critical role in controlling their CO_2_ adsorption at high pressures. The initial filling up of the micropores at low pressures is accompanied by multilayer formation at high pressures to fill all the available adsorption sites and voids.^[^ ^42^ ^]^ This is the case observed for the presented materials as well, which is evident from the shape of the isotherms (Figure 4a–c). At 0 °C and 30 bar, WPC600‐3, with the lowest surface area of 2332.90 m^2^ g^−1^ among all materials, adsorbed 14.57 mmoles of CO_2_ per gram of its weight (**Table** **2** and Figure 4a). The adsorption jumped ≈ twofold to 28.48 mmoles g^−1^ for WPC600‐4 with only a ≈1.45 times increase in the surface area (3377.94 m^2^ g^−1^). However, with only a slight increase (≈1.5 times) in the surface area, the subsequent materials WPC600‐5 (3487.60 m^2^ g^−1^) and WPC600‐6 (3686.10 m^2^ g^−1^) showed much higher adsorption of CO_2_ which amounts to an increase of ≈2.5 and ≈2.6 times as compared to WPC600‐3 (Figure 4a–c). The materials WPC600‐5 with a surface area of 3487.60 m^2^ g^−1^ showed 36.40 mmol g^−1^ of CO_2_ adsorption, whereas the material WPC600‐6 with a higher surface area of 3686.10 m^2^ g^−1^ showed higher CO_2_ adsorption of 37.47 mmol g^−1^. These materials do not possess a significantly large difference in their surface area, and hence their CO_2_ adsorption was also marginally different. The above observation for high‐pressure CO_2_ adsorption is also consistent with the mesopore contents, including mesopore volume and mesopore area of the materials (Figure 4d). The linear fitting of the mesopore volume and mesopore surface area yielded reasonable *R* ^2^ values (0.9845 and 0.9809), indicating a good fit in the context of high‐pressure CO_2_ adsorption. The CO_2_ adsorption at 10 °C and 25 °C shows a similar trend between surface area and CO_2_ adsorption (Figure 4b,c).

**Table 2:** CO_2_ adsorption of the materials.

Material	0 °C		10 °C		25 °C	
	1 bar	30 bar	1 bar	30 bar	1 bar	30 bar
WPC600‐3	4.85	14.57	3.98	13.75	2.90	12.42
WPC600‐4	5.59	28.48	4.21	27.06	3.00	23.40
WPC600‐5	5.35	36.40	4.24	33.90	2.70	27.98
WPC600‐6	4.69	37.47	3.87	34.52	2.76	28.33

#### 3.1.2 Effect of Microporosity on Low‐Pressure CO_2_ Adsorption

Micropores are crucial for low‐pressure CO_2_ adsorption in porous materials. WPC600‐3 showed a large content of microporous surface area (≈75% of the total 2332.90 m^2^ g^−1^) and hence a reasonable CO_2_ adsorption of 4.85 at 0 °C and 1 bar is recorded (Table 1 and 2). The next two materials WPC600‐4 and WPC600‐5, as discussed previously, possess considerably large surface areas, and even though their overall contribution from micropore area (40.8% and 25% of their total surface area) is smaller than WPC600‐3, their CO_2_ adsorption values at 0 °C and 1 bar are larger (5.59 and 5.35 mmol g^−1^, respectively) as compared to those of other carbon materials. It should be mentioned that WPC600‐6 showed the lowest CO_2_ adsorption of 4.69 mmol g^−1^ at 0 °C and 1 bar among all materials due to its very minimal contribution from micropores (13.3%) despite its largest specific surface area.

#### 3.1.3 Heat of Adsorption (*Q*_st_)

The temperature has a profound effect on CO_2_ adsorption of porous materials, which generally show an inverse relationship.^[^ ^44^ ^]^ It can be observed from Figure 4a–c that all materials perform better for CO_2_ adsorption at 0 °C while the least performance is observed at 25 °C. This illustrates the exothermic nature of the adsorption process using the current carbon materials.^[^ ^45^ ^]^ The increased motion of the CO_2_ molecules at high temperatures results in decreased CO_2_ adsorption.^[^ ^46^ ^]^ The adsorption isotherm at different temperatures also allows us to gauge the interactions between the adsorbent and the adsorbate. The quantitative factor for this type of analysis is the heat of adsorption (*Q*_st_), the value of which was obtained using Clausius Clapeyron's equation (Figure 4e). At the initial loading of CO_2_, the *Q*_st_ value of all materials lies in the range 20.27–27.70 kJ mol^−1,^ which suggests that the interactions between the material's surface and CO_2_ involve physical forces. A slight decrease in the *Q*_st_ value at higher CO_2_ loading can occur due to the multilayer adsorption of CO_2_ on the surface of the materials.^[^ ^46^ ^]^ Furthermore, the irregular shape of the curves is attributed to the heterogeneous surface of the materials.^[^ ^40^ ^]^ Overall, a low *Q*_st_ value is desirable as it allows for easier CO_2_ desorption from the surface of the materials and thus helps to lower the energy requirement for materials regeneration and reuse. A comparison of the CO_2_ adsorption of the presented materials was made with the previously reported materials^[^ ^29^, ^34^, ^35^, ^43^ ^]^ (Figure 4f), which showed a reasonably high performance of our materials.

#### 3.1.4 CO_2_ Recyclability, Dynamic Adsorption Experiments and CO_2_/N_2_ Selectivity

**Figure 5:**
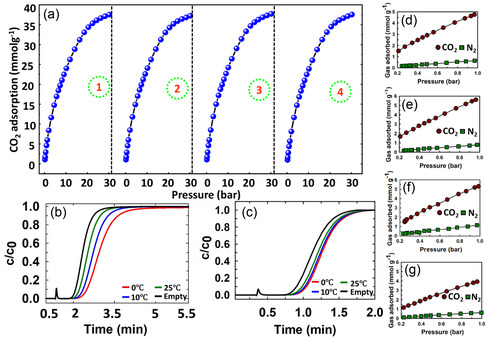
a) CO_2_ recyclability tests for WPC600‐6, b, c) CO_2_ breakthrough curves using 5% CO_2_ in helium balance and 1% CO_2_ in air balance, respectively, for WPC600‐6, and d–g) CO_2_ and N_2_ adsorption isotherms of WPC600‐3,4,5,6 (0 °C/1 bar) used for CO_2_/N_2_ selectivity calculations.

From a practical application point of view, an adsorbent should last for several cycles. Since current adsorbents operate via physisorption, their structure is expected not to undergo significant changes under the set experimental conditions of pressure and temperature. To validate this, the optimized material, WPC600‐6, which showed the highest CO_2_ adsorption at 30 bar/ 0 °C (37.47 mmol g^−1^), was subjected to continuous cycling operation for four consecutive adsorption and desorption cycles (**Figure** **5**a). Starting at 37.46 mmol g^−1^ at the first cycle, the material went on to display 37.46, 37.46, and 37.25 mmol g^−1^ at the second, third, and fourth cycles. This implies a retention of 99.41% of the CO_2_ adsorption capacity, which indicates excellent stability and reusability. The excellent cyclic stability ensures that the material can be used in industrial applications with minimal degradation over time, providing reliable and sustained CO_2_ capture performance, which holds significant implications for enhancing both the economic and energy efficiency of the overall process flow.

The dynamic breakthrough experiments were performed to understand the time‐dependent CO_2_ adsorption behavior of WPC600‐6. The corresponding breakthrough curves for CO_2_ adsorption (5% CO_2_ + helium balance and 1% CO_2_ + air balance) at 0 °C, 10 °C and 25 °C are presented in Figure 5b,c. It can be observed that the breakthrough time varies according to the temperature of adsorption and the type of gas mixture. Lower temperature of 0 °C provides a higher breakthrough time for either helium or air mixtures as compared to 10 °C and 25 °C, which suggests higher CO_2_ adsorption. The breakthrough point at either of the three temperatures is higher than an empty cell, which suggests CO_2_ adsorption by the material is occurring. In helium and air balance, the CO_2_ adsorption capacities at 0, 10, and 25 °C are 0.298, 0.251, and 0.198 mmol g^−1^ and 0.098, 0.094, and 0.089 mmol g^−1^, respectively. The difference in values could be attributed to the higher concentration of CO_2_ used in the helium balance (5%) as compared to the air balance (1%).

The CO_2_ capture in industrial post‐combustion operations requires its efficient separation from N_2_ in a 15:85 (CO_2_:N_2_) mixture. Therefore, any new adsorbent is benchmarked in terms of not only its CO_2_ adsorption capacity but also its ability for selective capture of CO_2_ over N_2_. The CO_2_ and N_2_ adsorption isotherms obtained for all four materials, WPC600‐3, 4, 5, and 6, for selectivity calculations, show a negligible adsorption of N_2_ as compared to CO_2_ (Figure 5d–g), which highlights their selective behavior for these gases. Quantitatively, as per the IAST method, the four materials showed reasonable CO_2_/N_2_ selectivity values of 32.53, 35.33, 25.41, and 25.54, respectively, which stands out as reasonable numbers when compared with other reported materials^[^ ^47^ ^]^ and also a handy number for practical CO_2_ separation. Only a marginal variation in the selectivity value of the materials could be attributed to their porosity difference.

### 3.2 Electrochemical Studies

**Figure 6:**
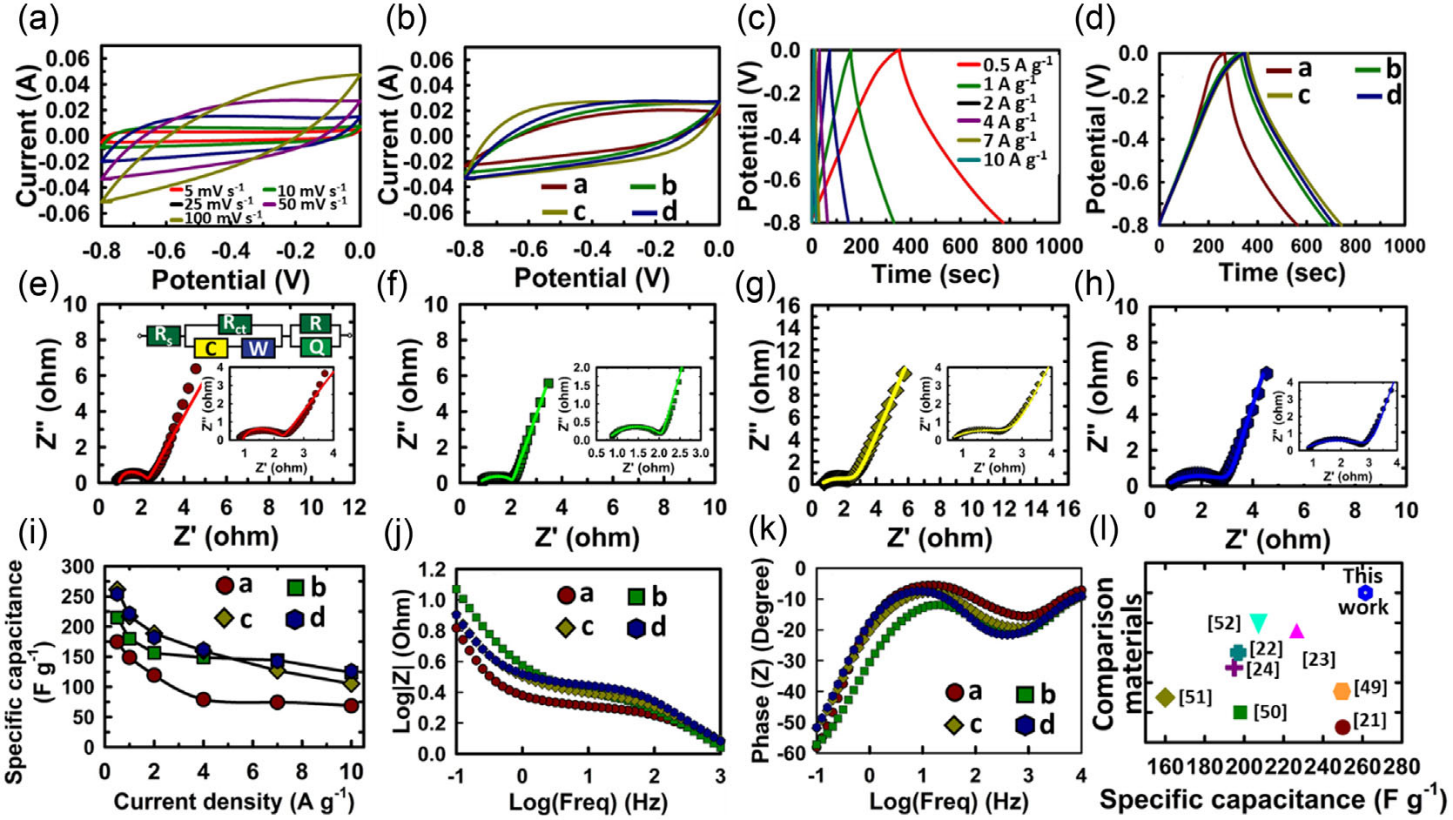
a) CV curves of WPC600‐5 at different scan rates, b) comparison of CV curves of WPC600‐3,4,5,6 at a scan rate of 50 mV s^−1^, c) GCD curves of WPC600‐5 at different current densities, d) comparison of GCD curves of WPC600‐3,4,5,6 at 0.5 A g^−1^, e–h) Nyquist plots WPC600‐3,4,5,6 within a frequency range of 1 Hz–10^3^ Hz, i) plot of the variation of specific capacitance (*C*_sp_) with current densities for WPC600‐3,4,5,6 (where a = WPC600‐3, b = WPC600‐4, c = WPC600‐5, and d = WPC600‐6), j) Bode magnitude plot, k) Bode phase plot, and l) comparison of *C*_sp_ of WPC600‐5 with the reported materials.^[^ ^21^, ^22^, ^23^, ^24^, ^49^, ^50^, ^51^, ^52^ ^]^

Micro and mesopores can play a critical role in controlling the electrochemical performance of porous carbon‐based materials. Therefore, the presented materials were evaluated for their electrochemical behavior using supercapacitor studies. The WPC600‐5 sample gave the best energy storage performance in a three‐electrode system, and its electrochemical properties in comparison with the other three materials are shown in **Figure** **6**.

#### 3.2.1 Three‐Electrode Setup

##### Cyclic Voltammetry (CV)

The CV curves of WPC600‐5 at different scan rates (5, 10, 20, 25, 50, 100 mV s^−1^) showed a quasirectangular shape at lower scan rates with a minor skewing at higher scan rates (Figure 6a). This exemplifies the low contact resistance along with good capacitive behavior of the material even at higher scan rates, which is possible due to a good mix of micro and mesopores and oxygen‐containing functional groups in the material. This behavior is typical for porous carbon materials and signifies that the charge storage primarily originates from the electric double‐layer capacitance (EDLC).^[^ ^45^ ^]^ It also indicates that the material allows for unrestricted charge transfer process at high scan rates, which is an attractive feature for application devices requiring faster charging and discharging.^[^ ^48^ ^]^ The CV curves for all four materials were also compared at 50 mV s^−1,^ and it was found that the shape of the curves tends to be more rectangular going from WPC600‐3 to WPC600‐6, which can be directly related to their mesopore content (Figure 6b). The CV curves of all four materials at various scan rates are presented in Figure S5 (Supporting Information).

##### Galvanostatic Charge/Discharge

The galvanostatic charge–discharge (GCD) curves of WPC600‐5 obtained at different current densities are shown in Figure 6c. A near triangular shape formed by the charge and discharge arms at all current densities, with a significantly larger area proportion at a current density of 0.5 A g^−1^ signifies the excellent supercapacitive behavior of the material. The specific capacitance value obtained from GCD is more reliable and practical, and WPC600‐5 displayed a high *C*_sp_ value of 261.50 F g^−1^ at a current density of 0.5 A g^−1,^ which lowered down to 104.98 F g^−1^ at a higher current density of 10 A g^−1^. In comparison (Figure 6d), the other three materials WPC600‐3,4,6 showed *C*_sp_ values of 174.85, 214.89, and 254.12 F g^−1^ at the current density of 0.5 A g^−1^. This indicates that micropores may not play a dominant role in electrochemical activity at low current densities, as WPC600‐3 have a considerably higher content of surface area arising from micropores (≈75 and ≈41%, respectively). In contrast, WPC600‐5 and WPC600‐6 exhibited much less micropore area content (25 and ≈13%), but their *C*_sp_ values were higher (261.5 and 254.12 F g^−1^). This shows that along with micropores, a significant amount of mesopores is crucial for enhancing electrochemical activity. Mesopores or micropores lying close to the border of 2 nm may participate in enhancing the mass transfer of the ions and lead to a higher *C*_sp_. Among WPC600‐5 and WPC600‐6, the former performed better at the lowest current density (261.5 F g^−1^); however, it was only marginally higher than WPC600‐5 (254.12 F g^−1^), and the latter gave higher performance under most of the current densities >0.5 A g^−1^. This was also confirmed from a comparison of the CD curves of all materials at 0.5 A g^−1,^ as shown in Figure 6d. All in all, an interplay of micro and mesopores is crucial in the presented porous carbon‐based materials to achieve an optimal value of *C*_sp_. The CD curves of all four materials at various scan rates are presented in Figure S6 (Supporting Information).

##### Electrochemical Impedance Spectroscopy (EIS)

The EIS spectra (an equivalent electric circuit) shown in Figure 6e–h present a near‐straight line at low frequency, signifying the Warburg element (*W*) and low ion diffusion resistance.^[^ ^53^ ^]^ In comparison, at higher frequencies, a low combined series resistance (*R*_s_), which involves the intrinsic resistance of electrode materials, ionic resistance of the electrolyte, and the contact resistance between the electrode and current collector, and a small semicircle, indicative of the charge transfer resistance (*R*_ct_), are observed.^[^ ^54^ ^]^ The smallest charge transfer resistance (*R*_ct_) gives the highest conductivity and best ionic diffusion on the carbon surface, leading to high *C*_sp_.^[^ ^55^ ^]^ Among all four materials, WPC600‐5 showed the lowest value of *R*_ct_ and hence the highest *C*_sp_ (**Table** **3**). A Bode plot is another method to evaluate the EIS behavior of the electroactive material. In practice, it helps determine the bandwidth of a supercapacitor and its ability to work with signals that comprise a specific frequency.^[^ ^56^ ^]^ A phase angle value between −90° to 90° gives a nonideal or mixed conductivity of the system. Bode plots based on the dependence of the complex impedance and phase angle with frequency values of the material are shown in Figure 6j,k. Impedance decreased at low frequencies but remained almost constant within the frequency range of 0–2 Hz, which indicated EDLC behavior. A phase angle of ≈−90° at the low‐frequency region is attributed to the EDLC behavior of the material. In the present case, the phase angle obtained was –60°, which is a good indication for the double layer phenomenon.^[^ ^56^ ^]^ A graphical representation of the variation of *C*_sp_ at various current densities in all materials is shown in Figure 6i. A comparative analysis of the *C*_sp_ of reported materials with WPC600‐5 in KOH electrolyte is shown in Figure 6l which confirmed the superior performance of the material reported in this work.

**Table 3:** The specific capacitance and EIS data of the materials.

Material	Specific capacitance, *C*_sp_ [F g^−1^] at various current densities [A g^−1^]						EIS parameters	
	0.5	1	2	4	7	10	*R*_*s*_ [ohm]	*R*_ct_ [ohm]
WPC600‐3	174.85	148.83	119.25	79.00	74.37	68.52	0.720	1.32
WPC600‐4	214.89	179.70	156.25	148.58	144.38	123.75	0.542	2.31
WPC600‐5	261.50	216.82	188.70	159.51	126.87	104.98	0.929	1.32
WPC600‐6	254.12	221.57	182.20	162.01	142.63	126.25	0.603	2.64

#### 3.2.2 Two‐Electrode Setup

**Figure 7:**
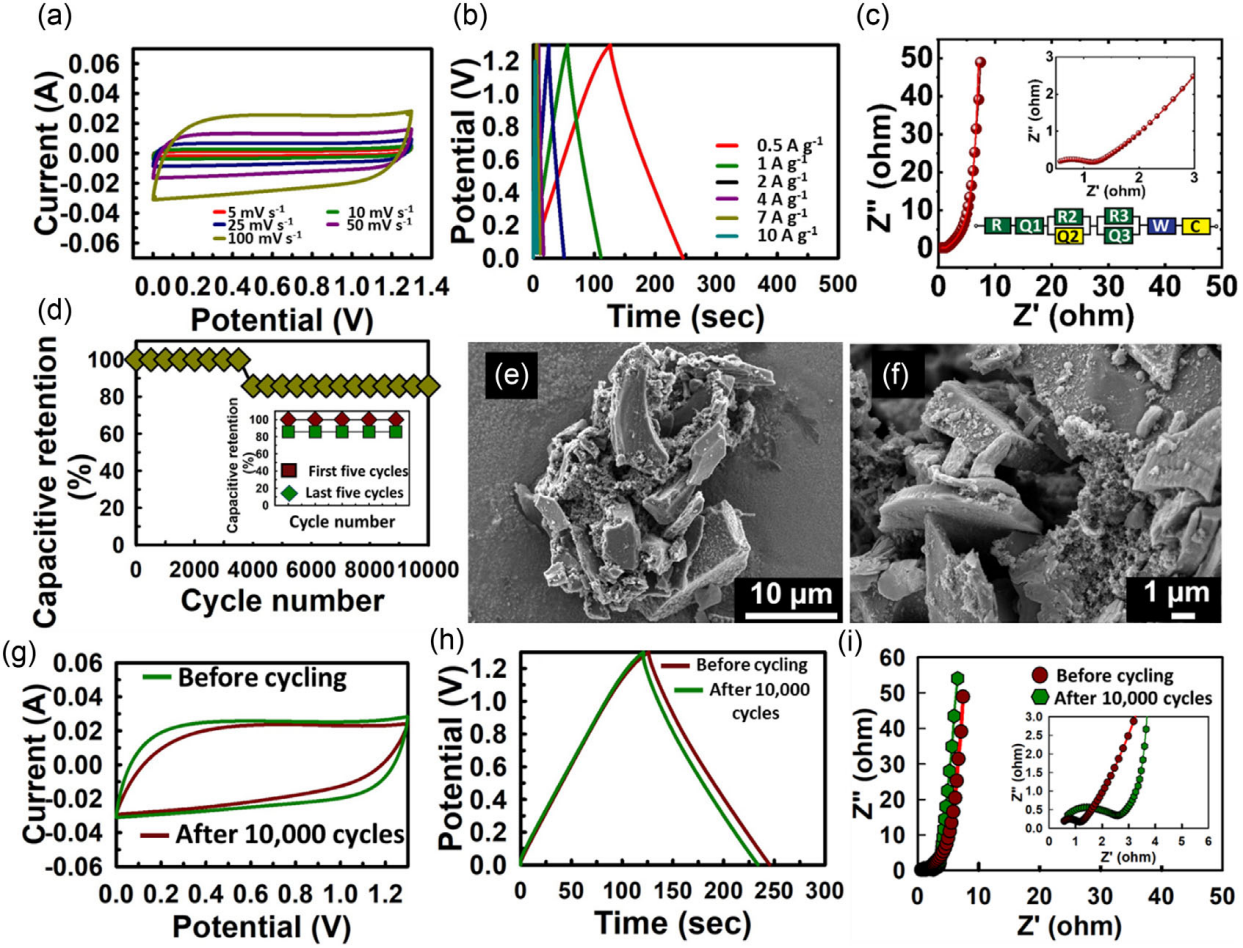
Two electrode electrochemical evaluation. a) CV curves of WPC600‐5 at different scan rates, b) GCD curves of WPC600‐5 at different current densities, c) Nyquist plot within a frequency range of 1 Hz–10^3^ Hz, and d) cycling stability performance over 10 000 cycles at a current density of 5 A g^−1^ (inset of Figure 7d gives the capacitive retention of WPC600‐5 in first five cycles (red) and last five cycles (green)), e–f) SEM images after 10 000 cycles, g) CV, h) GCD, and i) EIS of WPC600‐5 sample before and after 10 000 cycles.

Owing to the superior electrochemical performance of WPC600‐5, a two‐electrode symmetric supercapacitor was designed, and the obtained electrochemical properties are shown in **Figure** **7**. The CV curve retained its near‐rectangular shape even at scan rates as high as 100 mV s^−1^, which showed that the charge storage primarily occurred through a double‐layer mechanism (Figure 7a). The shape of the CV curves remains unaltered even at 100 mV s^−^^1^ attributed to the excellent stability of the material, fast charge transfer capability, and low equivalent series resistance. The GCD curves were used for evaluating the *C*_sp_ of WPC600‐5. The symmetrical triangular shape of GCD curves suggests the durable nature of the electrode during the charging and discharging cycles (Figure 7b). The calculated *C*_sp_ values of the material are 91.67 F g^−1^, 84.62 F g^−1^, 77.54 F g^−1^, 64.60 F g^−1^, 59.23 F g^−1^, and 46.15 F g^−1^ at respective current densities of 0.5 A g^−1^, 1 A g^−1^, 2 A g^−1^, 4 A g^−1^, 7 A g^−1^, and 10 A g^−1^. Furthermore, EIS was performed to further evaluate the electrochemical behavior of the materials and the obtained Nyquist plot, analyzed between a frequency range of 1 Hz–10^3^ Hz, is shown in Figure 7c. The charge transfer resistance (*R*_ct_) and intrinsic resistance (*R*_*s*_) were found to be 8.99 Ω and 0.371 Ω. The cyclic performance analysis for 10 000 cycles at a current density of 5 A g^−1^ revealed that the material maintains a 100% capacitive retention for up to 4000 cycles, followed by a capacitive retention of 87.5% till 10 000 cycles (Figure 7d). The inset in Figure 7d shows the cyclic stability of WPC600‐5 material with excellent capacitance retention of 100% for the first 5 cycles and capacitive retention in the last 5 cycles measured at 5 A g^−1^. The sudden dip in *C*_sp_ could be attributed to several reasons, including physical degradation, pore structure changes, or electrolyte‐related issues. To validate this, the post‐cycling SEM images, CV, CD, and impedance data were also recorded. The SEM images (Figure 7e,f) show disintegration of larger particles of carbon into smaller ones and deposition of tiny particles on the surface, which may have originated from potassium present in the electrolyte. The slight change in CV and CD curves (Figure 7g,h) is bound to happen after the cycling operation. The semicircle in the Nyquist plot (Figure 7i) has increased considerably after the cycling test, which indicates a higher *R*_ct_. Hence, the performance of WPC600‐5 is limited by fine deposits on the pores of the carbon surface, leading to lowered capacity after 10 000 cycles. The energy density and power density of the material were obtained as 18.33 Wh kg^−1^ and 2274.94 W kg^−1^ at 7 A g^−1^ and the Ragone plot of the material is shown in Figure S7, Supporting Information. Overall, the material displayed reasonable electrochemical performance in a two‐electrode system.

## 4 Conclusions

Nanoporous carbon was successfully synthesized from WWCs and used for the dual purpose of supercapacitor electrodes and CO_2_ adsorbent. A tailored porosity in the synthesized materials was attained by adjusting the carbonization temperature and the KOH impregnation ratio. A remarkably high surface area of 3686.10 m^2 ^g^−1^ and a high pore volume of 1.88 cm^3^ g^−1^ were achieved for the optimized nanoporous biocarbon sample derived from WWCs. It was also possible to tune the content of micro and mesopores and the surface oxygen functional groups (nitrogen and oxygen). The synthesized materials displayed outstanding performance in CO_2_ adsorption, wherein 5.59 mmoles and 37.47 mmoles of CO_2_ per gram of the adsorbent at 0 °C/1 bar and 0 °C/30 bar were recorded. As an electroactive material, the optimized nanoporous biocarbon achieved a good capacitive performance of 261.5 F g^−1^ and 91.67 F g^−1^ at a current density of 0.5 A g^−1^ in 3 M KOH electrolyte for three and two electrode setups, respectively. Reasonable energy and power densities of 18.33 Wh kg^−1^ and 2274.94 W kg^−1^ were also recorded, along with 87.5% capacity retention after 10 000 cycles. The suggested strategy of solid‐state KOH activation of wood chips promotes a remarkable foundation for the large‐scale application of sustainable activated porous carbons in gas capture and the fabrication of futuristic portable low‐cost supercapacitors.

## 5 Experimental Section

5.1

5.1.1

##### Chemicals

KOH, 98% hydrochloric acid (HCl), deionized (DI) water, AB, N‐methyl‐2‐pyrrolidone (NMP), and polyvinylidene fluoride (PVDF) were purchased from a different supplier and used as such without any further purification.

##### Material/s Synthesis

**Scheme 1:**
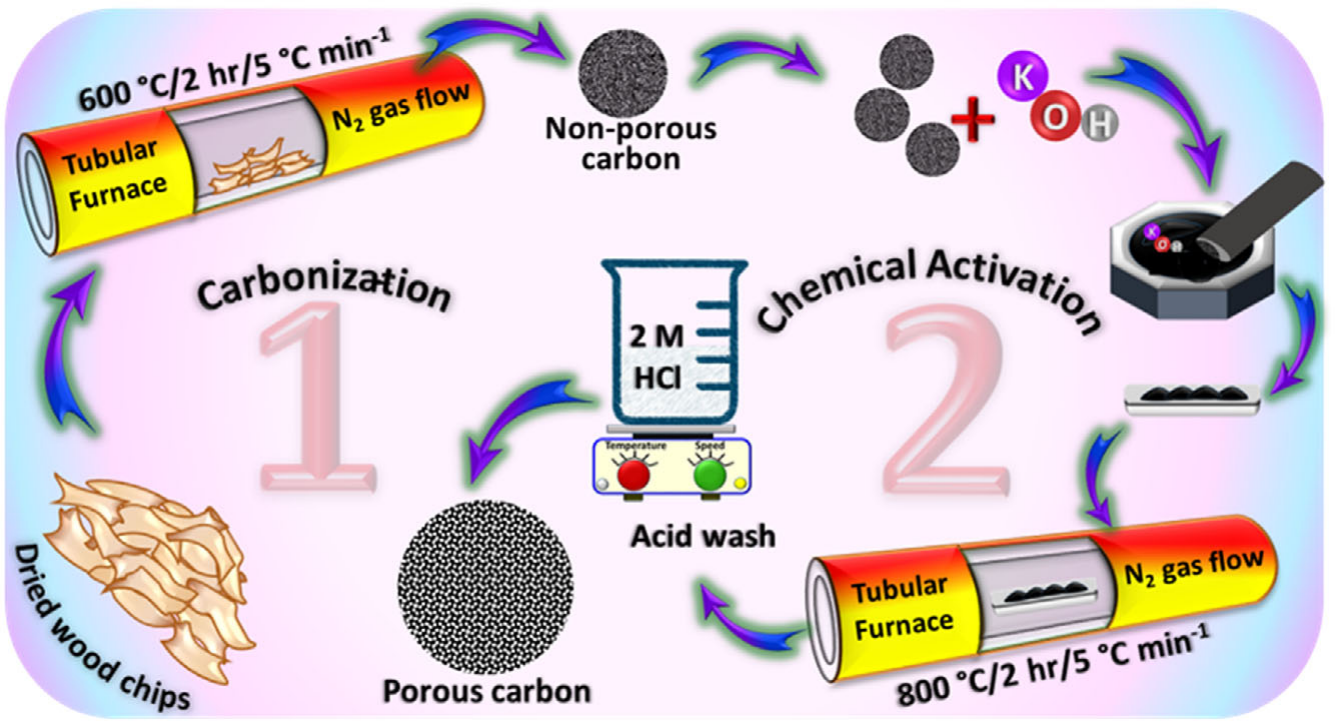
Schematic representation of the synthesis of materials.

WWCs were collected from the local council and processed via cleaning and drying prior to experimental synthesis. In detail, WWCs were initially washed with DI water to eliminate adhering debris, which was followed by overnight drying at 100 °C in a convection air oven. The dried WWCs were crushed into smaller pieces, finely ground, and sieved to obtain a uniform size of 125 μm. It is known that the complete carbonization of biopolymers, including cellulose, hemicellulose, and lignin, occurs beyond 500 °C and it leads to the formation of aromatic carbon structures.^[^ ^25^ ^]^ The sieved WWCs were carbonized under an inert atmosphere into nonporous carbons (NPCs) by keeping them for 2 h at three different temperatures of 400 °C, 500 °C, and 600 °C attained by using a ramping rate of 5 °C min^−1^. Various NPCs were synthesized by varying the carbonization temperature. The obtained NPCs are denoted as NPC‐T where T denotes the carbonization temperature. These NPCs were further chemically activated with KOH. In detail, 1 g of each NPC was physically mixed with 4 g KOH to obtain a uniform mixture, which was then subjected to carbonization at 800 °C for 2 h, with a ramping rate of 5 °C min^−1^. This was followed by 2 M HCl washing to neutralize potassium and other inorganic species and free up the pores. These species were then washed away with DI water using vacuum suction, and the materials dried overnight in a convection air oven at 100 °C. The yield of porous carbon after chemical activation lies in the range of 300–350 mg. These materials are denoted as WPCT‐X, where *T* denotes the temperature and *X* represents the amount of KOH added for the solid‐state activation in grams. A schematic for the synthesis of materials is presented in **Scheme** **1**.

##### Physicochemical Characterization of the Materials

A PANalytical Empyrean XRD instrument was used to collect the powder‐ray diffraction (PXRD) profile of the materials. The experimental setup includes X‐ray generation at 40 kV and 40 mA from Cu Kα1 (*λ* = 1.5406 Å) and Kα2 (*λ* = 1.5444 Å) radiation sources, and the PXRD patterns were recorded in the wide‐angle 2*θ* region of 10°–60° by using a step size of 0.006°. The porosity and surface area were analyzed using Micromeritics ASAP 2420 analyses‐based N_2_ sorption, wherein the initial degassing of materials at 200 °C under constant vacuum was followed by sorption measurements with N_2_ gas carried out at a temperature of −196 °C. The Brunauer–Emmett–Teller (BET) equation was used for the calculation of the specific surface area of the materials. Total pore volume was obtained from a single‐point adsorption at *p*/*p*_o_ of 0.99, and micropore contributions were obtained via the t‐plot method. The pore size was analyzed by using the NLDFT method. FTIR spectra were recorded using a PerkinElmer instrument over a wavenumber range from 400 to 4000 cm^−1^, with 4 cm^−1^ resolution and 32 scans per sample. JEOL JSM‐7900F scanning electron microscope operating at a voltage of 2 kV was used for measuring the surface morphological features of the materials. JEOL JEM‐2100F transmission electron microscope (TEM) was used to record the TEM imaging. XPS analysis was performed in a Thermo Scientific K‐alpha XPS instrument using a Monochromated Al Kα (E photon = 1486.7 eV) radiation source at a pressure of 1 × 10^−9^ Torr, and the high‐resolution scans were collected using 50 eV pass energy with 0.1 eV resolution. The quantity of C, H, and N in the composite materials was determined by using a CHNS analyzer.

##### Supercapacitor Evaluation

The three‐electrode electrochemical behavior of materials in 3 M KOH was investigated on a CH1760C electrochemical workstation, in which porous carbon, platinum (Pt), and saturated calomel electrode serve as working, counter electrode, and reference electrodes, respectively. The working electrode slurry was prepared using 65% of porous carbon, 25% acetylene black (AB), and 10% PVDF in a solution of N‐methyl pyrrolidone (NMP). Subsequently, this slurry was spread across an area of 1 cm^2^ on a nickel foam and then pressed using a roller presser and dried under vacuum at 60 °C for 12 h. A potential range of −0.8 V–0 V was chosen for CV measurements at scan rates between 5 mV s^−1^ to 100 mV s^−1^. Various current densities ranging from 0.5 A g^−1^ to 10 A g^−1^ were chosen for GCD analysis. EIS measurements were done within a range of 1 Hz–10^3^ Hz. The specific capacitance (*C*_sp_) of the prepared electrodes was calculated using GCD curves as per the following Equation (1)

(1) $$C_{\text{sp}}=\frac{I\Delta t}{m\Delta V}$$

where *I* denotes discharge current density (A), ΔV is the potential window, Δt represents the discharge time in seconds, and *m* represents the mass of the active material. Two working electrodes and a filter paper as an electrolyte separator membrane soaked in 3 M KOH in a CR2032 coin cell were used to study electrochemical behavior on a symmetric two‐electrode cell configuration. Three electrode as well as two electrode assemblies are demonstrated in Figure S1, Supporting Information. The *C*_sp_, specific energy density (*E*), and specific power density (*P*) were calculated via Equation ((2), (3))–(4 **)**;^[^ ^26^ ^]^

(2) $$C_{\text{sp}}=\frac{2*I*\Delta t}{m*\Delta V}$$

(3) $$E=\frac{1000*C\text{sp}*\Delta V^{2}}{2*3600}$$

(4) $$P=\frac{3600*E}{\Delta t}$$

##### CO_2_ Adsorption Experiments

A micrometric high‐pressure volumetric analyzer instrument was used for testing the CO_2_ adsorption of the materials. The materials were initially degassed under vacuum at ≈200 °C for 12 h to eliminate moisture and impurities. The CO_2_ adsorption was analyzed at 0 °C, 10 °C, and 25 °C within a pressure range of 0–30 bar. Isosteric heat of adsorption (*Q*_st_) values were obtained using the Clausius–Clapeyron equation (Equation (5)).

(5) $$\text{ln}\;p=\frac{-Q_{\text{st}}}{\text{RT}+\text{ln}C}$$

where *p*, R, T, and *C* denote the pressure, ideal gas constant, temperature, and a constant, respectively. A graph drawn between ln p versus 1/*T*, called the adsorption isostere, can be used to obtain values of *Q*_st_ from the slope of the graph. The linear fitting of the mesopore volume and mesopore surface area with high‐pressure CO_2_ adsorption at 30 bar was performed in the Origin software by using the linear fitting equation of *y* = *mx* + *C* (*m* = slope of the line, C = Y‐intercept at *X* = 0).

To evaluate the recyclability, the optimized material WPC600‐6 was subjected to four consecutive cycles of adsorption (30 bar/0 °C) and desorption (3 h/200 °C).

The CO_2_/N_2_ selectivity of materials was evaluated by using the CO_2_ and N_2_ adsorption isotherms data obtained at 0 °C and 1 bar. The adsorption isotherms were fitted using the dual‐site Langmuir–Freundlich model as per Equation (6).

(6) $$y=\frac{a_{1}b_{1}x^{c_{1}}}{1+b_{1}x^{c_{1}}}+\frac{a_{2}b_{2}x^{c_{2}}}{1+b_{2}x^{c_{2}}}$$

where *y* is the amount adsorbed (mmol g^−1^), *x* is the pressure in bar, *a* is the saturation capacity in mmol g^−1^, *b*_*i* (1 and 2)_ is the Langmuir parameter in bar^−1^, and *c*_*i* (1 and 2)_ is the Freundlich parameter for dual sites.

Later, the ideal adsorption solution theory (IAST) method was used for calculating CO_2_/N_2_ selectivity as per Equation (7).^[^ ^27^ ^]^ The CO_2_ and N_2_ fractions were taken as 15% and 85%, which is the typical composition of the flue gas.

(7) S= q1/q2p1p2

Where *S* is the selectivity coefficient, $q_{1}$ is the amount of CO_2_ adsorbed (mmol g^−1^), $q_{2}$ is the amount of N_2_ adsorbed (mmol g^−1^), $p_{1}$ is the the partial pressure of CO_2_ (kPa), and $p_{2}$ is the the partial pressure of N_2_ (kPa).

Dynamic CO_2_ adsorption studies were carried out using a dilute stream of CO_2_ (5%) in helium balance and also dilute CO_2_ (1%) in air balance at 0, 10, and 25 °C at 1 bar in a BELCAT II catalytic analyzer. A 4 cm long and 1.5 cm wide fixed bed column was packed with the optimized adsorbent WPC600‐6 and plugged on the ends using glass wool. Prior to the measurements, the material was degassed under vacuum at 250 °C for 2 h. The cooled down material was subjected to breakthrough analysis.

## Conflict of Interest

The authors declare no conflict of interest.

## Author Contributions

**Jibi Kunjumon**: conceptualization, methodology, software, formal analysis, writing—original draft; **Ajanya Maria Ruban**: SEM and CO_2_ data curation; **Harleen Kaur**: TEM data curation; **Davidson Sajan**: writing—review and editing; **Sanje Mahasivam** and **Vipul Bansal**: XPS data curation; **Gurwinder Singh**: conceptualization, formal analysis, visualization, supervision, writing—review and editing; **Ajayan Vinu**: conceptualization, resources, supervision, project administration, funding acquisition, writing—review and editing.

## Supporting information

Supplementary Material

## Data Availability

The data that support the findings of this study are available from the corresponding author upon reasonable request.
