# Supplementary material for: Functionalized Nanoporous Biocarbon with High Specific Surface Area Derived from Waste Hardwood Chips for CO2 Capture and Supercapacitors
Source: Small Sci. 2025 Jun 24;5(9):2500174. doi: 10.1002/smsc.202500174 (PMC12412513; doi:10.1002/smsc.202500174)
Supplement: Supplementary file 1 — Supplementary Material [file SMSC-5-2500174-s001.pdf]

**Supplementary information**

**Functionalized nanoporous biocarbon with high specific surface area derived from waste hardwood chips for $CO_2$ capture and supercapacitors**

**Table S1:** Specific surface area($SA_{BET}$), micropore area ($SA_{micro}$) and micropore volume ($V_{micro}$) of porous carbon carbonized at 400, 500 and 600 °C

| Material | $SA_{BET}$ | $SA_{micro}$ | $V_{micro}$ |
|---|---|---|---|
| WPC400-4 | 3257.09 | 2240.50 | 0.4024 |
| WPC500-4 | 3366.26 | 1454.04 | 0.5911 |
| WPC600-4 | 3377.94 | 1380.2 | 0.4797 |

**Table S2:** EDX elemental composition of the materials

| Sample code | Carbon | | Oxygen | |
|---|---|---|---|---|
| | Mass % | Atomic % | Mass % | Atomic % |
| WPC600-3 | 98.58 | 98.93 | 1.42 | 1.07 |
| WPC600-4 | 99.06 | 99.29 | 0.94 | 0.71 |
| WPC600-5 | 99.04 | 99.28 | 0.96 | 0.72 |
| WPC600-6 | 98.71 | 99.03 | 1.29 | 0.97 |

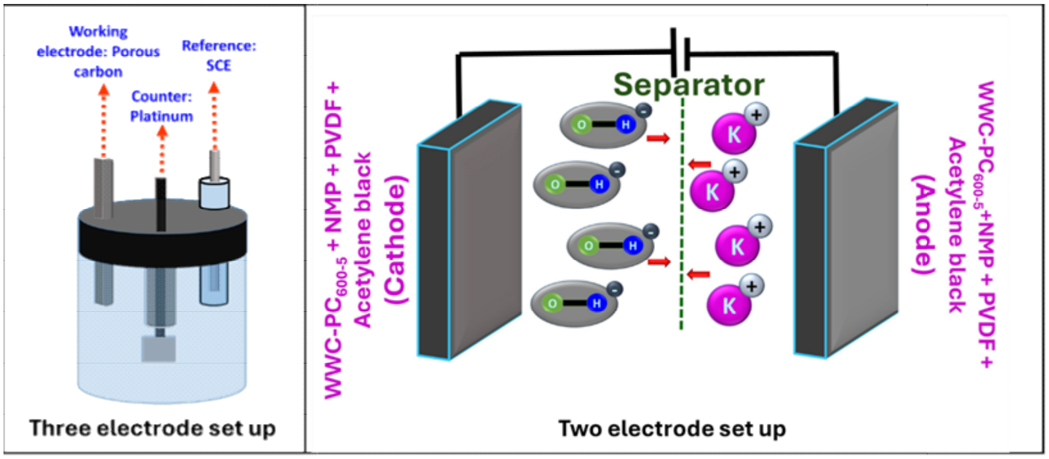


**Figure S1:** Three-electrode and two-electrode setup for electrochemical measurements.

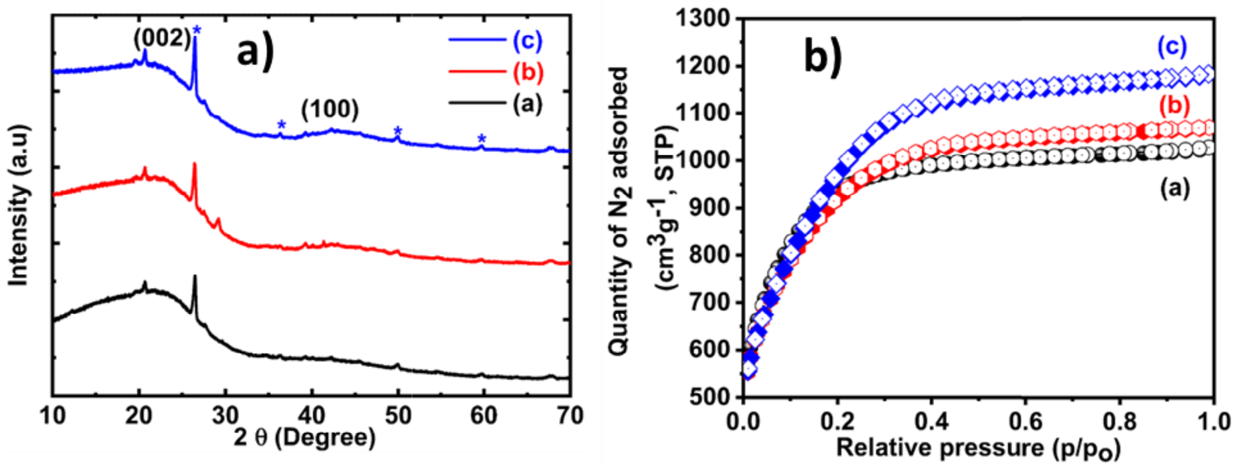


**Figure S2: a)** XRD patterns of NPCs (where a = NPC-400,b = NPC-500, c = NPC-600)* = biomass minerals and **b)** $N_2$ sorption isotherms of a) WPC400-4, **b)**WPC500-4, and **c)**WPC600-4

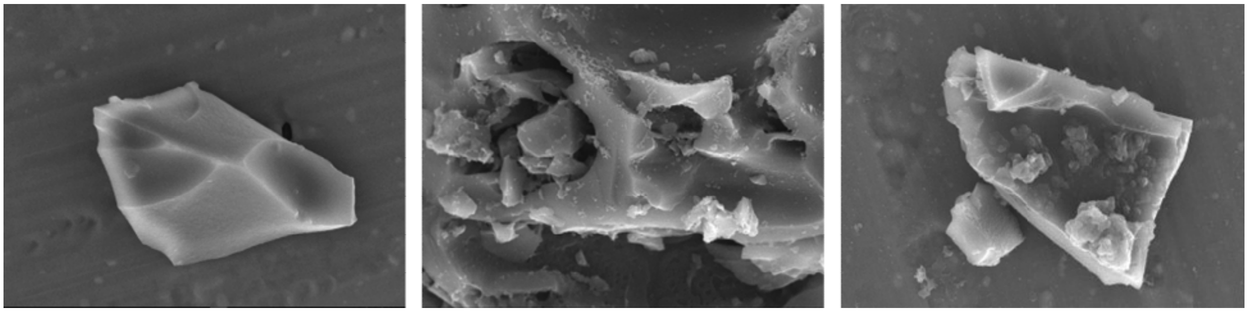

**Figure S3**: SEM images of a) WPC400-4, **b)**WPC500-4, and **c)**WPC600-4

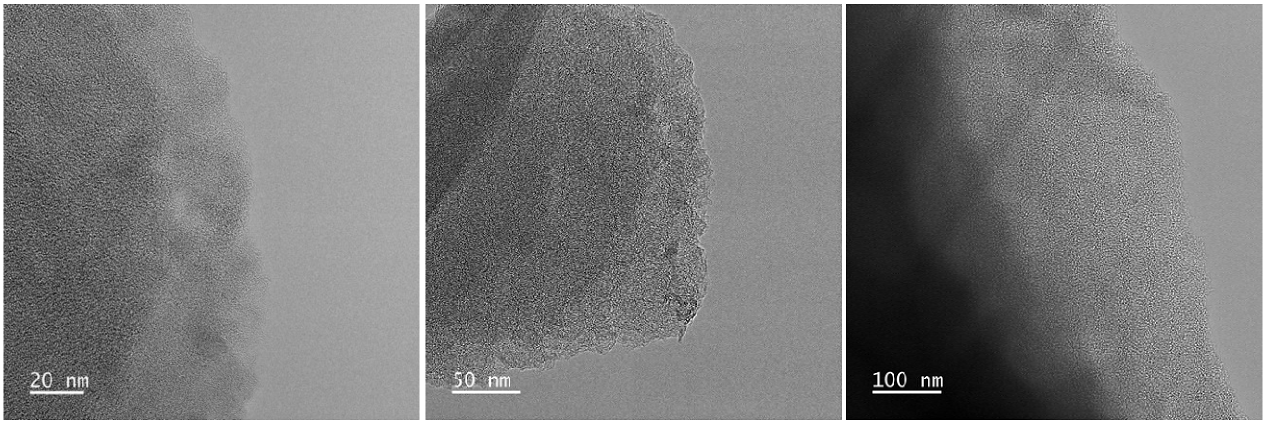


**Figure S4**: HRTEM images of WPC600-6

**Table S3**: Comparison – Activation temperature, $CO_2$ uptake capacity among carbon precursors

| Carbon precursor | Activation Temperature (°C) | Surface area ($m^2 g^{-1}$) | $CO_2$ Adsorption Conditions (Temp °C/Pres bar) | $CO_2$ Uptake Capacity ($mmol g^{-1}$) | Ref. |
|---|---|---|---|---|---|
| Alligator weed | 800 | 3106 | 0/30 | 30.4 | [43b] |
| Aundodonax | 700 | 3298 | 0/30 | 30.2 | [35] |
| Benzimidazole-LinkedPolymers | 700 | 3240 | 0/30 | 25.7 | [43d] |
| Walnut shell | 800 | 2497 | 0/30 | 18.2 | [43f] |
| Grape marc | 800 | 2473 | 0/30 | 26.8 | [29] |
| Lotus seed | 900 | 2230 | 0/30 | 26.4 | [34] |
| MOF-505 | - | 2804 | 0/30 | 26.1 | [43a] |
| N- doped Arundo donax | 500 | 1420 | 25/30 | 18.2 | [43e] |
| phenol–formaldehyde resin-derived carbons | - | - | 25/15 | 8.5 | [43c] |

**Notes**: The reference numbers are the same as per Figure 4f in the main manuscript

**TableS4:** Comparison – Surface area, specific capacitance among biomass-sources-based activated carbon

| Biomass precursor | Surface area ($m^2 g^{-1}$) | Electrolyte | Specific capacitance ($F g^{-1}$) | Current density ($Ag^{-1}$) | Ref. |
|---|---|---|---|---|---|
| N-doped carbon aerogel derived from banana | 1414.97 | KOH | 178.9 | 1 | [22] |
| Endothelium corneum gigeriaegalli | 2149.9 | 6M KOH | 198.0 | 1 | [50] |
| Corn Silk Derived Nanoporous Carbon | 1246 | 6M KOH | 160 | 1 | [51] |
| Taro stems | 1012 | 6M KOH | 236.4 | 0.1 | [23] |
| Hibiscus sabdariffa fruits | 1720 | 2M KOH | 194.50 | 0.5 | [24] |
| Porous carbon aerogels derived from chitosan | 2435.2 | 6M KOH | 197 | 0.2 | [22] |
| Nitrogen-doped potatowaste | 1052 | 2M KOH | 255 | 0.5 | [49] |

| | | | | | |
|---|---|---|---|---|---|
| Enteromorphaprolifera | 3332 | 6M KOH | 210 | 3 | [52] |

**Notes**: The reference numbers are the same as per Figure 6l in the main manuscript

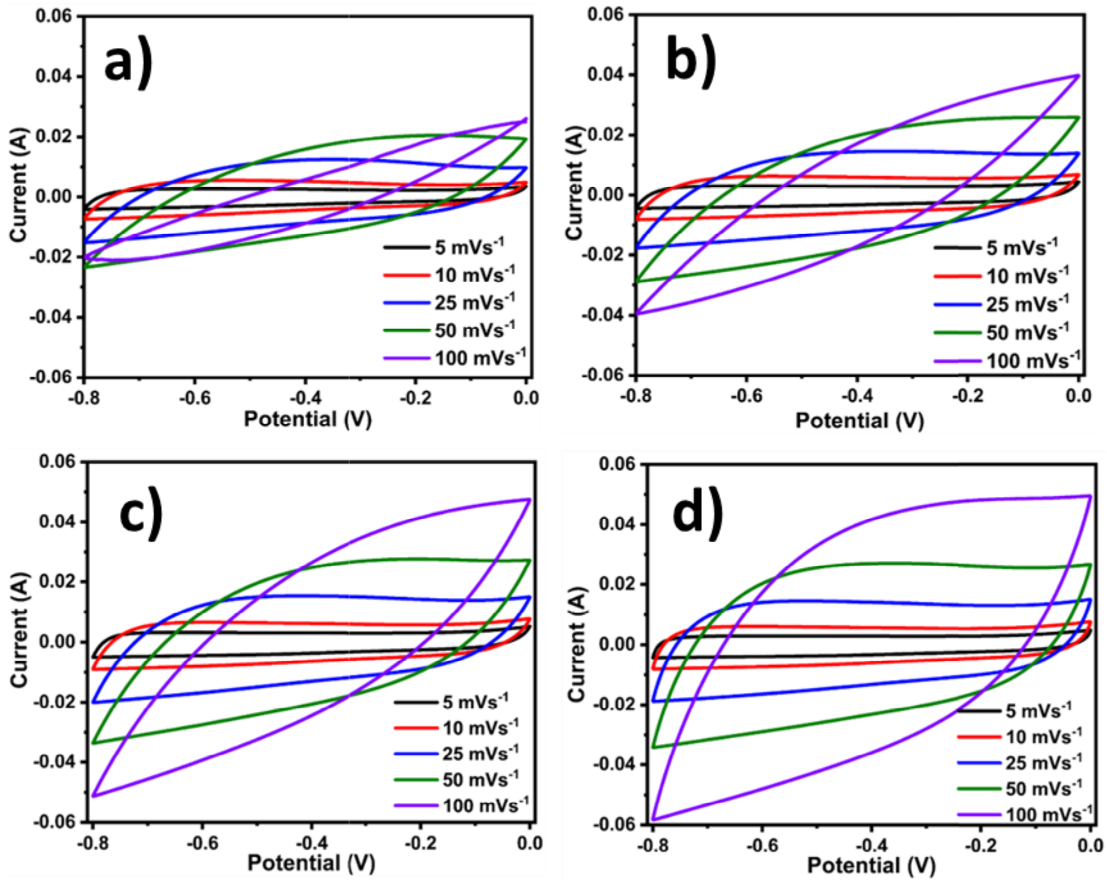


**Figure S5**: CV curves of a) WPC600-3, **b)**WPC600-4, and **c)**WPC600-5, and d) WPC600-6

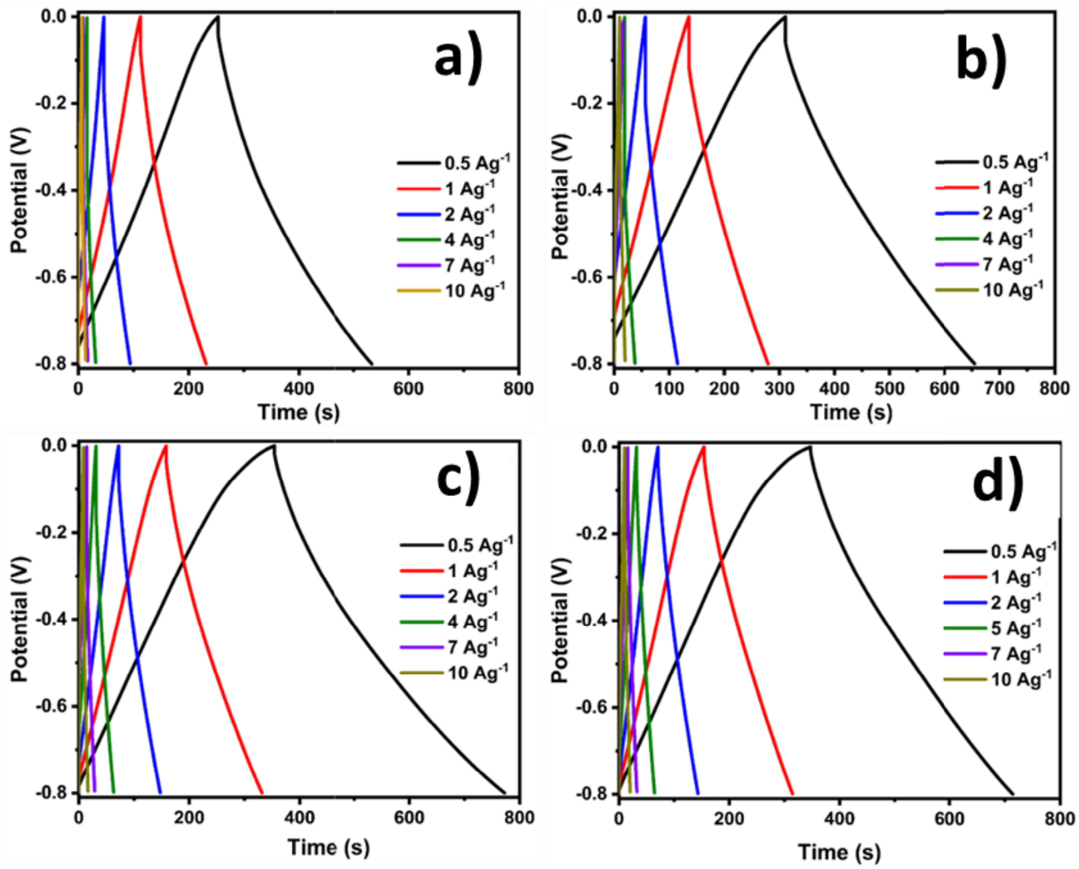


**Figure S6**: CD curves of a) WPC600-3, **b)**WPC600-4, and **c)**WPC600-5, and d) WPC600-6

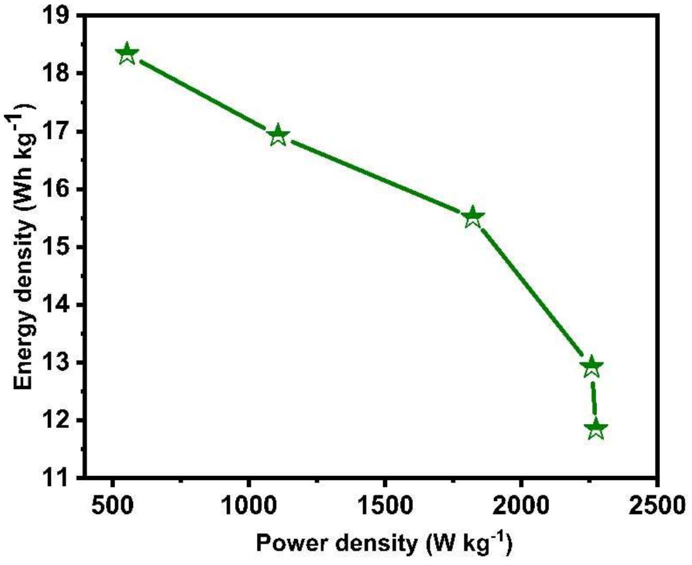


**Figure S7**: The Ragone plot of WPC600-5
